# Mental health and fatigue status of the medical workforce during the COVID-19 outbreak in the Yangzhou city, China

**DOI:** 10.3389/fpsyt.2022.1018069

**Published:** 2022-10-17

**Authors:** Xiaojia Tang, Yunlong Pei, Xin Wang, Li Jiang, Peipei Liu, Yingzhu Chen, Zhaoxiang Meng

**Affiliations:** ^1^Department of Rehabilitation Medicine, Northern Jiangsu People’s Hospital Affiliated to Yangzhou University, Yangzhou, Jiangsu, China; ^2^Department of Rehabilitation Medicine, Clinical Medical College, Yangzhou University, Yangzhou, Jiangsu, China; ^3^Department of Critical Care Medicine, The Affiliated Hospital of Yangzhou University, Yangzhou, Jiangsu, China; ^4^Department of Geriatric Neurologist, Clinical Medical College, Yangzhou University, Yangzhou, Jiangsu, China; ^5^Department of Neurology, Clinical Medical College, Yangzhou University, Yangzhou, Jiangsu, China

**Keywords:** COVID-19, anxiety, depression, fatigue status, medical workforce

## Abstract

**Background:**

When the coronavirus disease 2019 (COVID-19) erupted in Yangzhou, China, at the end of July 2021, medical workers in Yangzhou immediately joined the frontline for the fight against the pandemic. This study aimed to identify the mental health and fatigue experienced by the medical workers in Yangzhou during the COVID-19 outbreak.

**Methods:**

We included 233 medical workers who participated in the front-line work for more than 1 month through the questionnaire, including doctors, nurses, medical technicians and medical students. The generalized anxiety disorder-7 (GAD-7), patient health questionnaire-9 (PHQ-9), and Fatigue self-assessment scale (FSAS) were administered to the participants and their responses were evaluated.

**Results:**

A total of 233 eligible questionnaires were received. Among them, 130 people (57.08%) were probably anxious and 141 (60.52%) people were clinically depressed. Poor sleep was considered an independent risk factor for anxiety (OR = 7.164, 95% CI: 3.365 15.251, *p* = 0.000) and depression (OR = 6.899, 95% CI: 3.392 14.030, *p* = 0.000). A high PHQ-9 score was considered an independent risk factor for general fatigue (OR = 1.697, 95% CI: 1.481 1.944, *p* = 0.000). Mental fatigue (OR = 1.092, 95% CI: 1.027 1.161, *p* = 0.005) and fatigue response to sleep/rest (OR = 1.043, 95% CI: 1.011 1.076 *p* = 0.008) were considered independent risk factors for general fatigue.

**Conclusion:**

Poor quality of sleep led to probable anxiety, depression, and general fatigue. Mental fatigue and fatigue response to sleep/rest were independent risk factors for depression, which merits attention for battling COVID-19.

## Introduction

Since the outbreak of coronavirus disease-19 (COVID-19) in December 2019, the new coronavirus pneumonia has become a global public health emergency (1). After the outbreak in Wuhan was effectively controlled, provinces and cities of China performed normalized management activities for the pandemic, which effectively controlled the spread of the virus. Only a few areas had confirmed cases and were quickly under control. At the end of July 2021, the epidemic spread rapidly in Yangzhou, China, and the transmission chain was long. The first case of COVID-19 was diagnosed on July 28, and 94 cases were confirmed on August 2, just 3 days after the first case was diagnosed, then Yangzhou underwent a city-wide lockdown on 8.3. Apart from the typical symptoms caused by the Delta strain of this virus, specific local symptoms were observed (2). The virus spreads because of crowded gatherings in confined spaces, and the infected people are mainly middle-aged and elderly. The middle-aged and elderly people have poor awareness of prevention and many underlying diseases, thus leading to the development of severe infection easily. The Center for Disease Control and Prevention (CDC) has created many obstacles for conducting activity trajectory investigations and sub-close contact investigations on close contacts. For the first time, medical staff had to devote themselves to frontline work by blocking communities and cities, increasing the number of rounds of nucleic acid testing at the national level, and performing a round of screening to confirm the diagnosis and homebound population.

Severe acute respiratory syndrome coronavirus 2 (SARS-CoV-2), which is responsible for COVID-19, causes symptoms such as cough and fever, severe pneumonia, and death (3). Coronaviruses are enveloped, positive-stranded RNA viruses that belong to the Coronaviridae family and the order Nidovirales. SARS-CoV-2 is the seventh member of the coronavirus family (4). In addition, SARS-COV-2 variants such as Delta, Beta, Alpha, and Gamma strains have also been found in different countries (5). Additional mutations and increased virulence capability of the virus have caused people to experience different degrees of anxiety, stress, and other mental conditions. Previous studies have shown that infected patients are prone to develop psychological and sleep disorders (6, 7).

Excessive anxiety is likely to affect the ability of health care workers to pay attention, understand and make decisions when they encounter an outbreak (8). A study involving 2,089 health workers suggested that providing care to COVID-19 patients has a marked emotional impact, as 51.75% of the health workers developed clinical anxiety and 38.58% developed clinical depression (9). Quarantine management, long working hours, different guidance and safety measures, and the risk of infection have also caused psychological stress for medical workers. Şahan and Tangılntız found that when the trust in PPE is lower, healthcare worker anxiety is higher (10). Whether there is enough personal protective equipment (PPE) also makes medical workers anxious, PPE is considered to be an effective buffer against self-contagion and infection of others (11).

Due to the special nature of their work, medical workers have to participate in shift work, and they are also troubled by sleep disorders. The length of sleep and poor sleep quality can disrupt the normal circadian rhythm (12). Johnson et al. found that insomnia is associated with anxiety and depression, and may be a risk factor for their subsequent development. Additionally insomnia may be independent, potentially etiologically distinct, directional associations with anxiety disorders vs. depression (13).

Fatigue refers to the subjective feeling of physical fatigue, lack of strength or mental exhaustion, inability to concentrate, and behaviorally manifested as a decline in physical or mental activity, including physical fatigue and mental fatigue. Fatigue is a very common phenomenon or symptom, which can be seen not only in healthy people, but also in many diseases. It can also be caused by certain medical measures (such as cancer radiotherapy, chemotherapy, etc.), especially in sub-healthy people (14). Whether in their daily work or in the fight against the epidemic, medical staff need focused mental work and high-intensity physical activities, which will inevitably lead to fatigue. Fatigue is a frequently reported symptom in major depressive disorder, occurring in over 90% of patients. Clinical presentations of fatigue within major depressive disorder encompass overlapping physical, cognitive and emotional aspects. The symptom of fatigue in major depressive disorder both at baseline and as a residual symptom following treatment (15). The residual symptoms of fatigue after treatment in patients with severe depressive disorder also caused a serious clinical burden (16). A study in Pakistan proved that greater exhaustion, the greater family strain, and reduced feelings of protection impact on levels of anxiety among physicians (17). Increased clinical responsibility, the risk of infection and the possibility of infecting family members are the main reasons for medical workforce’s fear and anxiety (18).

Research on the correlation between fatigue, anxiety, and depression levels among medical workers is limited and rare. In this study, the anxiety, depression, and fatigue levels were evaluated based on GAD-7, PHQ-9, and FSAS scores to understand the extent of anxiety, depression, and fatigue experienced by the medical workforce during the COVID-19 pandemic in Yangzhou City.

## Materials and methods

### Participants

An online cross-sectional study was performed among the medical workforce in late August 2021 in Yangzhou City. Since Yangzhou City (Jiangsu Province) launched the first-level response to major public health emergencies in July 2021, the medical workforce in Yangzhou who had participated in epidemic prevention and control for more than 15 days have been included. A total of 233 medical workers were surveyed in this study, including 81 men (34.8%) and 152 women (65.2%). Thirty-five participants (15.0%) were over 40 years of age, 128 (54.9%) were married, 145 (62.2%) held a bachelor degree or below, 142 (60.9%) held a junior professional title, 60 (25.8%) held an intermediate professional title, 31 (13.3%) held senior professional titles, 25 (10.7%) held administrative positions at the same time, and 180 (72.3%) people slept well (62.2%).

The inclusion criteria for the study subjects were as follows: (1) Participation in the front-line epidemic prevention and control and (2) agree to participate in the study. The exclusion criteria included the following: (1) complicated with physical and mental diseases; (2) those who have not completed all scale measurements. In this cross-sectional survey, we received a total of 235 questionnaires, of which, 2 incomplete questionnaires were excluded. The remaining 233 questionnaires were complete. Informed consent of all study participants and the guidelines were followed as outlined in the Declaration of Helsinki.

### Clinical data collection

The questionnaire survey was conducted using the platform of Wenxianxing,^1^ which covered basic information such as age, marital status, occupation, title, and the GAD-7, PHQ-9, and FSAS scales. Complete questionnaires finished within 7 days from August 30 to September 6, 2021, were considered eligible and included in the following analysis.

### Assessment scale

Anxiety disorder can be divided into generalized anxiety disorder (GAD), panic disorder (PD), social anxiety disorder (SAD), post-traumatic stress disorder (PTSD) and so on, GAD is the most common form of expression (19). The GAD-7 consisted of 7 items, and it is a valid and efficient tool to screen for generalized anxiety disorder (GAD) as well as assess its severity in clinical practice and research (20). This scale used the following grading scheme: no anxiety: 0–4 Points, mild anxiety: 5–9 points, medium anxiety: 10–14 points, heavy anxiety: 15–21 points.

The Patient Health Questionnaire-9 (PHQ-9) was based on the 9 items of the Diagnostic and Statistical Manual of Mental Disorders developed by the American Psychiatric Association diagnostic criteria. It is a simple and effective self-rating scale used for depression. It shows good reliability and validity in the auxiliary diagnosis of depression and in the evaluation of the severity of symptoms (21). It used the following grading scheme: no depression: 0–4 points, mild depression: 5–9 Points, medium depression: 10–14 Points, heavy depression: 15–27 Points.

The fatigue self-assessment scale (FSAS) is a fatigue self-assessment scale developed in 2008 by Professor Tianfang Wang’s team using psychometric methods. This scale is composed of 23 projects, which can be used to assess the fatigue performance and the fatigue type of health and diseases crowd degree (including physical fatigue, mental fatigue, and fatigue effects of 3 factors) and characteristics (including fatigue response to sleep/rest of situational and the time of fatigue model three-factor) and the intervention effect of fatigue (11). The standard points of general fatigue are divided, and the specific grades and the corresponding score ranges are as follows: not obvious: 3 points, mild: 3–40 Points, medium: 41–80 Points, heavy: 81–100 Points.

The surveyors’ own evaluation of sleep was 1, 2, 3, 4, and 5 points: very poor, poor, average, good and very good. A score greater than or equal to 4 was classified as good sleep group, and a score less than 4 was classified as poor sleep group.

### Statistical analyses

Statistical Package for Social Science version 25.0 was utilized to analyze the data in this study. Descriptive statistics were used to review the properties of the study population. Categorical variables were presented *via* frequency and percentage. Continuous variables were presented via means and standard deviation (mean ± SD). Continuous variable data, in line with normal distribution, used independent sample *T*-test for single factor study, and select (mean ± SD); if it did not obey the normal distribution, select two independent samples of non-parameters. The test is a single-factor study, and the median (interquartile range) (interquartile range, IQR) was selected. Internal consistency reliability-based Cronbach’s alpha(Cronbach’s α) test, Kaiser-Meyer-Olkin Test of Sampling Adequacy(KMO) and Bartlett’s test of sphericity were used to estimate the scales’ construct validity and reliability. The risk factors of anxiety and depression were evaluated through binary logistics regression. *p* < 0.05 indicated that the difference was statistically significant.

## Results

### Prevalence of generalized anxiety disorder and depression

The GAD-7, Cronbach’s α value was 0.938, KMO value was 0.922 and the significance level of Bartlett’s test of sphericity at *p* < 0.000. The PHQ-9 score, Cronbach’s α value was 0.937, KMO value was 0.924 and the significance level of Bartlett’s test of sphericity at *p* < 0.000. The FASS scale, Cronbach’s α value was 0.931, KMO value was 0.939 and the significance level of Bartlett’s test of sphericity at *p* < 0.000. The above three scales have good reliability and validity.

Among the medical staff that participated in the survey, the GAD-7 score was 5.57 ± 4.78, and 103 (44.21%) were without anxiety, 90 (38.63%) had mild anxiety, 28 (12.02%) had moderate anxiety, and 12 (5.15%) had severe anxiety. The PHQ-9 score was 6.72 ± 5.78, and 92 (39.48%) were without depression, 91 (39.06%) had mild depression, 26 (11.16%) had moderate depression, and 24 (10.30%) had severe depression. The general fatigue score was 24.10 ± 24.38, and 53 (22.75%) did not experience fatigue, 129 (55.36%) had mild fatigue, 42 (18.03%) had moderate fatigue, and 9 (3.86%) had severe fatigue.

### Anxiety and depression

The non-anxiety group consisted of 103 (44.21%) individuals and the anxiety group included 130 (55.79%) individuals. Univariate factor analysis showed that marriage, job title, working-age, and sleep quality were suspicious risk factors for causing anxiety, and there were significant statistical differences between the two groups (all *p*<0.05) (Table 1). Incorporating the above risk factors into the binary logistics regression analysis revealed that poor sleep was an independent risk factor for causing anxiety (OR, 7.164; 95% CI, 3.365–15.251, *p* = 0.000) (Table 2).

**TABLE 1:** Univariate analysis of anxiety and depression among medical workforce.

Variables	Anxiety				Depression			
	Non-anxiety group	Anxiety group	X^2^/Z	*p*	Non-depression group	Depression group	X^2^/Z	*p*
**Sex**								
Male	35 (43.2%)	46 (56.8%)	0.05	0.823	25 (30.9%)	56 (69.1%)	3.862	0.049
Female	68 (44.7%)	84 (55.3%)			67 (44.1%)	85 (55.9%)		
**Age (y)**								
18–40	87 (43.9%)	111 (56.1%)	0.038	0.845	75 (37.9%)	123 (62.1%)	1.423	0.233
41–60	16 (45.7%)	19 (54.3%)			17 (48.6%)	18 (51.4%)		
**Marital status**								
Married	49 (38.3%)	79 (61.7%)	4.042	0.044	48 (37.5%)	80 (62.5%)	0.468	0.494
Single	54 (51.4%)	51 (48.6%)			44 (41.9%)	61 (58.1%)		
**Occupation**								
Doctor	36 (41.4%)	51 (58.6%)	2.968	0.397	29 (33.3%)	58 (66.7%)	8.017	0.046
Nurse	40 (51.9)	37 (48.1%)			40 (51.9%)	37 (48.1%)		
Medical technician	19 (38.0%)	31 (62.0%)			18 (36.0%)	32 (64.0%)		
Medical student	8 (42.1%)	11 (57.9%)			5 (26.3%)	14 (73.7%)		
**Title**								
Junior or lower	72 (50.7%)	70 (49.3%)	7.405	0.025	62 (43.7%)	80 (56.3%)	3.304	0.192
Intermediate	18 (30.0%)	42 (70.0%)			18 (30%)	42 (70%)		
Senior	13 (41.9%)	18 (58.1%)			12 (38.7%)	19 (61.3%)		
Work age(y), median (IQR)	4 (1–10)	2 (8–13)	–2.272	0.023	4 (1–11)	6 (1.5–12.5)	–1.074	0.283
**Education level**								
Undergraduate or lower	66 (45.5%)	79 (54.5%)	0.268	0.605	61 (42.1%)	84 (57.9%)	1.073	0.300
Graduate or higher	37 (42.0%)	51 (58.0%)			31 (35.2%)	57 (64.8%)		
Administrative position	11 (44.0%)	14 (56.0%)	0.000	0.982	8 (32.0%)	17 (38.0%)	0.657	0.418
Working days against the epidemic (d), median (IQR)	22 (15–30)	19 (24.5–30)	–0.575	0.565	23.5 (15.25–30)	23 (19–30)	–1.729	0.084
Sleep duration (h), median (IQR)	6 (6–7)	6 (6–7)	–1.854	0.064	6 (6–7)	6 (6–7)	–0.535	0.593
**Self-evaluation of sleep**								
Good sleep	31 (33.9%)	119 (66.1%)	34.151	0.000	53 (29.4%)	128 (70.6%)	33.387	0.000
Poor sleep	42 (79.2%)	11 (20.8%)			39 (73.6%)	14 (26.4%)		

**TABLE 2:** Binary logistics regression analysis of anxiety and depression among medical workforce.

	Anxiety				Depression			
	*p*	OR	95% CI		*p*	OR	95% CI for OR	
Poor sleep	0.000	7.164	3.365	15.251	0.000	6.899	3.392	14.030

The non-depression group consisted of 92 (39.48%) participants and the depression group consisted of 141 (60.52%) participants. Univariate factor analysis showed that sex, occupation, and sleep quality were suspicious risk factors for causing depression, and there were significant statistical differences between the two groups (all *p*<0.05) (Table 1). Incorporating the above risk factors into the binary logistics regression analysis showed that poor sleep was an independent risk factor for causing depression (OR, 6.899; 95% CI, 3.392–14.030, *p* = 0.000) (Table 2).

### Fatigue

Among those with overall fatigue, 53 (22.70%) did not have fatigue, 129 (55.40%) had mild fatigue, 42 (18.00%) had moderate fatigue, and 9 (3.90%) had severe fatigue. Univariate analysis showed that title, sleep duration, sleep quality, and GAD-7 and PHQ-9 scores were suspected risk factors for causing fatigue (all *p*< 0.05) (Table 3). The above suspicious risk factors were included in the orderly logistics regression analysis, which showed that PHQ-9 score was an independent risk factor for overall fatigue development (OR, 1.697; 95% CI, 1.481–1.944, *p* = 0.000) (Table 4).

**TABLE 3:** Univariate analysis of general fatigue among medical workforce.

	General fatigue					
	Non	Mild	Moderate	Severe	X/Z	*P*
**Sex**						
Male	13 (16.0%)	49 (60.5%)	16 (19.8%)	3 (3.7%)	3.252	0.354
Female	40 (26.3%)	80 (52.6%)	26 (17.1%)	6 (3.9%)		
**Age(y)**						
18–40	45 (22.7%)	109 (55.1%)	37 (18.7%)	7 (3.5%)	0.709	0.871
41–60	8 (22.9%)	20 (57.1%)	5 (14.3%)	2 (5.7%)		
**Marital status**						
Married	22 (17.2%)	72 (56.3%)	27 (21.1%)	7 (5.5%)	7.279	0.064
Single	31 (29.5%)	57 (54.3%)	15 (14.3%)	2 (1.9%)		
**Occupation**						
Doctor	14 (16.1%)	49 (56.3%)	18 (20.7%)	6 (6.9%)	15.174	0.086
Nurse	26 (33.8%)	38 (49.4%)	12 (15.6%)	1 (1.3%)		
Medical technician	8 (16%)	34 (68%)	7 (14%)	1 (2%)		
Medical student	5 (26.3%)	8 (42.1%)	5 (26.3%)	1 (5.3%)		
**Title**						
Junior or lower	38 (26.8%)	81 (57%)	21 (14.8%)	2 (1.4%)	17.583	0.007
Intermediate	10 (16.7%)	27 (45%)	17 (28.3%)	6 (10%)		
Senior	5 (16.1%)	21 (67.7%)	4 (12.9%)	1 (3.2%)		
Work Age(y), median (IQR)	4 (1∼9.5)	5 (1∼13)	8 (2∼14.25)	10 (4.5∼17.5)	7.795	0.050
**Education level**						
Undergraduate or lower	38 (26.2%)	75 (51.7%)	26 (17.9%)	6 (4.1%)	3.017	0.389
Graduate or higher	15 (17%)	54 (61.4%)	16 (18.2%)	3 (3.4%)		
Administrative position	4 (16%)	17 (68%)	4 (16%)	0 (0%)	2.513	0.473
Working days against the epidemic	30 (18.5∼32.5)	21 (15.5∼30)	26 (20∼31.25)	30 (20.5∼35)	7.273	0.064
Sleep duration	6 (6∼7)	6 (6∼7)	6 (6∼7)	5 (4∼6.5)	10.993	0.012
**Self-evaluation of sleep**						
Good sleep	30 (16.7%)	101 (56.1%)	40 (22.2%)	9 (5%)	23.321	0.000
Poor sleep	23 (43.4%)	28 (52.8%)	2 (3.8%)	0 (0%)		
GAD-7 score	1 (0∼3.5)	5 (3∼7)	9 (6∼13.25)	19 (18.5∼21)	90.095	0.000
PHQ-9 score	2 (0∼3)	6 (3∼8)	12.5 (9∼15.25)	25 (21∼27)	133.071	0.000

**TABLE 4:** Binary logistics regression analysis of general fatigue among medical workforce.

General fatigue						
		Regression coefficient	*P*	OR	95% CI for OR	
GAD-7 score		0.019	0.755	1.019	0.906	1.147
PHQ-9 score		0.529	0.000	1.697	1.481	1.944
Sleep duration		0.039	0.826	1.040	0.732	1.477
Self-evaluation of sleep	Good sleep	0.018	0.964	1.018	0.465	2.230
	Poor sleep	0.000				
Title	Junior or lower	0.061	0.897	1.063	0.420	2.691
	Intermediate	0.903	0.088	2.467	0.875	6.959
	Senior	0.000				

PF, Physical fatigue; MF, Mental fatigue; CF, Consequences of fatigue; RFSAR, Fatigue response to sleep/rest; SF, Severity of fatigue.

To explore the effects of fatigue-causing factors and characteristics of anxiety and depression experienced by the medical staff, the degree of fatigue (including physical fatigue (PF), mental fatigue (MF), and consequences of fatigue (CF)), characteristics of fatigue (fatigue response to sleep/rest (RFSAR), and situationality of fatigue (SF)) were analyzed by a single-factor conditional logistic regress analysis. The details of correlation among anxiety-, depression-, and fatigue-causing factors are presented in Table 5. The scores of PF, MF, CF, RFSAR, and SF in the anxiety group were significantly higher than those in the non-anxiety group with statistically significant differences (all *p* < 0.05). The scores of PF, MF, CF, RFSAR, and SF in the depression group were significantly higher than those in the non-depression group with statistically significant differences (all *p* < 0.05). PF, MF, CF, RFSAR, and SF scores were included in the binary logistics regression analysis for anxiety, and no fatigue-causing factor was an independent risk factor for anxiety (all *p* > 0.05). PF, MF, CF, RFSAR, and SF scores were included in the binary logistics regression analysis of depression, and we found that MF (OR, 1.092; 95% CI, 1.027–1.161, *p* = 0.005) and RFSAR scores (OR, 1.043; 95% CI, 1.011–1.076, *p* = 0.008) were independent risk factors for depression (Table 6).

**TABLE 5:** Univariate analysis of fatigue factor among anxiety and depression.

	Anxiety				Depression			
	Non-anxiety group	Anxiety group	X^2^/Z	*P*	Non-depression group	Depression group	X^2^/Z	*P*
PF	6.25 (0–18.75)	25 (18.75–43.75)	70.978	0.000	0 (0–12.5)	25 (18.75–43.75)	102.20	0.000
MF	6.25 (0–18.75)	28.13 (18.75–50)	72.258	0.000	0 (0–12.5)	31.25 (25–50)	112.08	0.000
CF	4.17 (0–16.67)	25 (20.83–50)	83.302	0.000	0 (0–11.46)	25 (20.83–50)	111.18	0.000
RFSAR	0 (0–12.50)	25 (9.38–50)	60.793	0.000	0 (0–0)	25 (12.5–50)	83.47	0.000
SF	25 (5.00–40.00)	55 (25–75)	39.669	0.000	22.5 (0–40)	55 (25–75)	44.61	0.000

PF, Physical fatigue; MF, Mental fatigue; CF, Consequences of fatigue; RFSAR, Fatigue response to sleep/rest; SF, Severity of fatigue.

**TABLE 6:** Binary logistics regression of fatigue factor among anxiety and depression.

	Anxiety				Depression			
	*P*	OR	95% CI		*P*	OR	95% CI	
PF	0.968	0.999	0.958	1.042	0.594	1.015	0.961	1.072
MF	0.543	1.015	0.968	1.063	0.005	1.092	1.027	1.161
CF	0.061	1.054	0.998	1.115	0.572	1.02	0.952	1.093
RFSAR	0.079	1.019	0.998	1.041	0.008	1.043	1.011	1.076
SF	0.293	1.007	0.994	1.021	0.577	1.005	0.988	1.022

PF, Physical fatigue; MF, Mental fatigue; CF, Consequences of fatigue; RFSAR, Fatigue response to sleep/rest; SF, Severity of fatigue.

**FIGURE 1:**
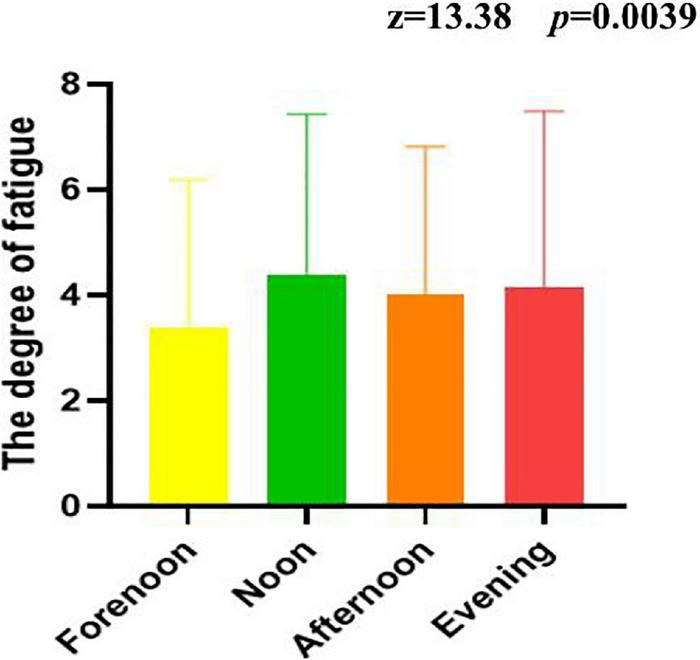
The time pattern of fatigue of medical workforce.

Figure 1 shows the time pattern of fatigue of the medical workforce. It can be seen that the extent of fatigue was the highest at noon, followed by night, afternoon, and morning. A non-parametric test indicated that the extent of fatigue in the morning, middle, afternoon, and night was statistically significant (*z* = 13.38, *p* = 0.0039).

## Discussion

We investigated the parameters of mental wellbeing (indexed by the pattern of psychological anxiety and depression) and fatigue in healthcare workers that worked on the frontline during the COVID-19 pandemic in Yangzhou City. A total of 233 healthcare workers completed the questionnaire with quality assurance. Importantly, the study assessed mental health and fatigue among these healthcare workers during the COVID-19 pandemic that may guide the effect of the small-scale COVID-19 epidemic or outbreak on medical personnel in parts of China or other countries so as to provide logistical support, psychological counseling, and other countermeasures.

Among the medical workforce who participated in our study, 152 (65.2%) of healthcare workers were women and 81 (34.8%) of healthcare workers were men. A high women participation rate was found in other studies, and the percentages were similar (22). In a meta-analysis that included 12 descriptive studies on the psychological effect of the SARS-CoV-2 crisis on the frontline medical workforce, women and nurses were more likely to experience high-intensity stress, anxiety, depression, fatigue, and sleep disorders (23). In our study, the number of women with anxiety was more than that of men but the difference was not statistically significant; this may be related to the small sample size and that women also tend to develop depression to a higher extent than men and this difference is statistically significant. It may be related to the particularity of the women’s physiological structure and functions; most women experience physical discomfort such as abdominal bloating, fatigue, breast tenderness, etc. during menstruation and they are also prone to negative emotions such as low mood and irritability (24).

Poor sleep was an independent risk factor for developing anxiety and depression in our study. Somnipathy refers to the various dysfunctions pertaining to the sleep-waking cycle. Insomnia is a highly prevalent, often debilitating, and economically burdensome form of sleep disturbance caused by various situational, medical, emotional, environmental, and behavioral factors (25). Poor sleep quality is a common complaint, with up to 15% of adults suffering from chronic sleep disorders (26). Mental disorder can also induce sleeping problems, which, in turn, can contribute to more mental disorders. Also, high levels of anxiety and depression are often evident in insomniacs, and there is also a high incidence of sleep disorders in people with anxiety or depressive disorders (27). Sleep is also associated with anxiety and depression. Cox and Olatunji proposed that defects in the executive function and dysregulated hypothalamic-pituitary-adrenal (HPA) axis activity likely mediate the relationship between sleep disturbance and anxiety-related disorders. Insufficient sleep can increase cortisol production and a decrease in total sleep time is associated with a smooth decline in cortisol levels and an increase in cortisol levels at night (28). Chronically high cortisol levels and/or inadequate cortisol response to acute stress may lead to anxiety disorders. Interestingly, Léger et al. (29) found that insomniacs have more work-related accidents and a higher rate of absenteeism. Insomnia patients more so than good sleepers avoid social activities to a large extent and have difficulty concentrating because of feelings of fatigue and sleepiness (30). From the perspective of psychoimmunology, ensuring a good sleep for the medical staff can not only improve their mental state but also improve their immune system (31). This can protect frontline health professionals, so that they turn their energy to complete daily diagnosis and treatment, thus rendering our health systems to be more secure.

Fatigue is a subjective feeling, including physical and mental fatigue, and behaviorally manifested as a decrease in physical or mental activity. Fatigue is a common phenomenon or symptom that can be observed in healthy people, those with several diseases, and those undergoing medical treatment (such as radiotherapy, chemotherapy, etc.), and is particularly the most common manifestation in subhealthy people. Because fatigue can affect people’s quality of life to different degrees, with the change in Modern Medical and the gradual emphasis on quality of life, the research on fatigue has attracted considerable attention. The commonly used fatigue scales mainly consist of fatigue scale (FS-14), fatigue severity scale (FSS), fatigue assessment scale (FAS), fatigue impact scale (FIS), and profile of fatigue-related symptoms (PFRS) (32). These scales can be used to evaluate the extent of fatigue and the changes in functional status caused by fatigue (33). These scales have their own strengths but they lack structural validity and have certain limitations in evaluating severe fatigue. Our research adopted the fatigue self-assessment scale (FSAS), which is in line with Chinese humanistic characteristics and language habits, by quantifying general fatigue (GF), physical fatigue (PF), mental fatigue (MF), consequences of fatigue (CF), severity of fatigue (SF), and fatigue response to sleep/rest (RFSAR) to analyze the fatigue experienced by the medical staff during this pandemic.

Fatigue is a common symptom of major depressive disorder, occurring in more than 90% patients (15). Fatigue in case of major depression is associated with depressive and insomnia symptoms (34). Research by Dornonvil le De La Cour et al. suggested that general fatigue, mental fatigue, and reduced motivation may be associated with depression (35). Another study found that depression was independently associated with mental fatigue, reduced motivation, physical fatigue, and reduced activity but not general fatigue (36). Mental fatigue was more common than physical fatigue in studies on acquired brain injury, and Buunk et al. found that mental but not physical fatigue was independently associated with poor long-term outcomes after subarachnoid hemorrhage (SAH). Consistent with the findings of previous studies, our study showed that a high PHQ-9 score was related to GF. The five fatigue factors PF, MF, CF, RFSAR, and SF were all possible risk factors for anxiety and depression, and MF and RFSAR were independent risk factors for depression.

The severity fluctuates throughout the course of the day, and the pattern and degree of fatigue varies with each disease or condition. In a fatigue study performed on oncology patients found that higher morning and evening fatigue trajectories were associated with younger age, as well as higher scores on state and trait anxiety, depression, and sleep disturbance (37). On the other hand, the high evening fatigue pattern was associated with anxiety and the high morning pattern was associated with anxiety and depression in people with AIDS or HIV (38). Medical workforce wore airtight protective clothing to conduct nucleic acid sampling outdoors at temperatures up to 40 degrees Celsius, and work for 4–6 h. The ward staff of patients diagnosed with COVID-19 is rotated once every 6 h and once every 4 days. In our study, midday fatigue was the highest among healthcare workers, possibly related to the end of morning work and shift schedules.

Nevertheless, there are several strengths and key limitations of our study. To the best of our knowledge, this is the first study to assess the correlation among fatigue, anxiety, and depression in healthcare workers by using the FSAS scale during the COVID-19 outbreak. The deficiencies of this study are as follows: (1) Our sample size is not large enough; (2) the evaluation of sleep could have been performed in many aspects; and (3) only cross-sectional investigation was performed without psychological intervention for the frontline medical workforce. Our future studies will be addressing these limitations.

## Conclusion

To summarize, our study highlighted that poor quality of sleep probably led to the development of anxiety, depression, and general fatigue. Mental fatigue and fatigue response to sleep/rest were independent risk factors for fatigue, which merits attention during the battle against COVID-19.

## Data availability statement

The raw data supporting the conclusions of this article will be made available by the authors, without undue reservation.

## Author contributions

All authors made a significant contribution to the work reported, whether that was in the conception, study design, execution, acquisition of data, analysis and interpretation, and in all these areas, took part in drafting, revising and critically reviewing the article, gave final approval of the version to be published, agreed on the journal to which the article had been submitted, and agreed to be accountable for all aspects of the work.

## Footnotes

1
www.wjx.cn/
